# Dysfunction of Human Estrogen Signaling as a Novel Molecular Signature of Polycystic Ovary Syndrome

**DOI:** 10.3390/ijms242316689

**Published:** 2023-11-24

**Authors:** Clémentine Marie, Alice Pierre, Anne Mayeur, Frank Giton, Raphael Corre, Michaël Grynberg, Joëlle Cohen-Tannoudji, Céline J. Guigon, Stéphanie Chauvin

**Affiliations:** 1 Université Paris Cité, CNRS, Inserm, Unité de Biologie Fonctionnelle et Adaptative, 75013 Paris, France; clementine.marie@etu.u-paris.fr (C.M.); alice.pierre@inserm.fr (A.P.); raphael.corre@u-paris.fr (R.C.); michael.grynberg@aphp.fr (M.G.); joelle.cohen-tannoudji@u-paris.fr (J.C.-T.); celine.guigon@univ-paris-diderot.fr (C.J.G.); 2 Service de Médecine de la Reproduction et Préservation de la Fertilité, Hôpital Antoine Béclère, 92140 Clamart, France; anne.mayeur@aphp.fr; 3 AP-HP, Pôle Biologie-Pathologie Henri Mondor, Inserm IMRB U955, 94010 Créteil, France; frank.giton@u-pec.fr

**Keywords:** granulosa cells, polycystic ovary syndrome, 17β-estradiol, steroids, follicular fluid

## Abstract

Estradiol (E2) is a major hormone-controlling folliculogenesis whose dysfunction may participate in polycystic ovary syndrome (PCOS) infertility. To determine whether both the concentration and action of E2 could be impaired in non-hyperandrogenic overweight PCOS women, we isolated granulosa cells (GCs) and follicular fluid (FF) from follicles of women undergoing ovarian stimulation (27 with PCOS, and 54 without PCOS). An analysis of the transcript abundance of 16 genes in GCs showed that androgen and progesterone receptor expressions were significantly increased in GCs of PCOS (by 2.7-fold and 1.5-fold, respectively), while those of the steroidogenic enzymes *CYP11A1* and *HSD3B2* were down-regulated (by 56% and 38%, respectively). Remarkably, treatment of GC cultures with E2 revealed its ineffectiveness in regulating the expression of several key endocrine genes (e.g., *GREB1* or *BCL2*) in PCOS. Additionally, a comparison of the steroid concentrations (measured by GC/MS) in GCs with those in FF of matched follicles demonstrated that the significant decline in the E2 concentration (by 23%) in PCOS FF was not the result of the E2 biosynthesis reduction. Overall, our study provides novel hallmarks of PCOS by highlighting the ineffective E2 signaling in GCs as well as the dysregulation in the expression of genes involved in follicular growth, which may contribute to aberrant folliculogenesis in non-hyperandrogenic women with PCOS.

## 1. Introduction

Polycystic ovary syndrome (PCOS) is the most frequent endocrinopathy, affecting 5–10% of women of reproductive age [1]. PCOS is a heterogeneous disorder, which is defined by the presence of at least two of the following features after the exclusion of other endocrinopathies: hyperandrogenism, oligo-anovulation and polycystic ovarian morphology. Abnormal folliculogenesis is considered as the common characteristic of PCOS, with an excess of an initial recruitment of primordial follicles along with an arrest of follicular development at the stage when the selection of follicles occurs. Consequently, the ovaries of PCOS women contain a large number of small and premature antral follicles. These preantral follicles showed a higher survival capacity [2,3], with granulosa cells (GCs) responding prematurely to the luteinizing hormone (LH) [4] and exhibiting a higher proliferation rate than normal [5]. However, when examining follicles at a later stage of development, such as preovulatory follicles obtained from women undergoing in vitro fertilization (IVF) protocols, several studies reported a significant increase in the apoptosis capacity of GCs in PCOS [6,7,8,9,10].

In the follicular fluid (FF) of polycystic ovaries, the estradiol (E2) concentration was reported being below the reference concentration found in the dominant follicles of healthy women [11,12]. FF results principally from GC and thecal cell secretions and provides a microenvironment for the growth and maturation of oocytes and follicular cells. Its composition in cytokines, growth factors, lipids, polysaccharides, metabolites, hormones and micro-RNAs have been associated with oocyte competence and maturation as well as GCs functionality [13,14,15,16,17,18]. Therefore, the default in the FF composition may impair oocyte quality [19] and the outcomes of in vitro fertilization [19,20]. Few studies have compared the steroid profiles of FF between PCOS and control women using liquid chromatography–tandem mass spectrometry, enabling the comparison of multiple steroids within the same sample. They have confirmed the hyperandrogenic nature of PCOS FF as well as the lower concentration of E2 when compared to the controls [21,22,23]. However, the molecular basis of the E2 concentration decline in PCOS FF is still under debate. It has been postulated that the lower E2 concentration could result from ovarian steroidogenesis dysfunction and/or proteins that are present in the FF, like 5α-androstane-3,17-dione, that would inhibit aromatase (CYP19A1) activity and prevent the efficient conversion of thecal androgens to estrogens [24,25]. Still, it is necessary to explore this area in detail, and notably to define whether E2 biosynthesis is really disturbed in GCs and could account for its decline in the FF of PCOS women.

Reduction in the E2 concentration in the FF of PCOS women may influence the level of expression of critical genes involved in GCs function that would contribute to follicle growth arrest. In line with this hypothesis, a decrease in the mitogen-activated protein kinase and Wnt/β-catenin pathways, as well as an increase in inflammation-related gene expression, has been reported in a transcriptome analysis of GCs from follicles of PCOS women (24–26). Still, these observations remain carried out on small cohorts of few studies.

Within antral follicles, E2 is locally produced by GCs upon follicle-stimulating hormone (FSH) stimulation to strengthen follicular growth and maturation [26]. Studies on primates showed that E2 could exert atretogenic effects on dominant preovulatory follicles by reducing both GCs viability and steroid secretion [27]. Since E2 is important for regulating follicle growth as well as for the selection and atresia of subordinate follicles [26], any alteration in the E2 concentration and/or in the activity of estrogenic signaling mediated by estrogen nuclear receptor ERα and ERβ (with four isoforms in GCs [28]) may lead to PCOS dysfunctions. Resolving these questions would help to clarify the role of E2 in impaired follicular selection in PCOS.

In this study, we revealed a striking difference in E2 signaling activities between control and PCOS women and identified significant deregulations in mRNA expressions of specific genes in GCs of PCOS follicles. In addition, we found that the difference in steroid concentrations in the FF of PCOS women may not be totally attributed to steroidogenesis dysfunction.

## 2. Results

### 2.1. Patients Characteristics

A total of 27 patients who met the PCOS criteria and 54 patients with non-ovarian indications for undergoing IVF were participants in this study. All PCOS participants were characterized by a polycystic ovarian morphology and ovulatory dysfunction (oligo/amenorrhea), without any sign of clinical hyperandrogenism. This PCOS phenotype is the least represented of the three others (~20% [29]), which justifies the lower number of the PCOS patients than the non-PCOS ones. PCOS patients and non-PCOS patients followed the same GnRH antagonist stimulation protocol, which is recommended for PCOS patients, normal or poor responder patients, with regard to improved safety and equal efficacy [30]. All patients showed equivalent serum E2 levels (2528 ± 4.97 vs. 2219 ± 20.87 pg/mL, respectively) before hCG administration; these concentrations were below risks for ovarian hyperstimulation syndrome (˂3000 pg/mL) [31,32] (Table 1). The mean age and body mass index (BMI) slightly differed between PCOS patients and non-PCOS patients; PCOS patients were significantly younger and had significantly higher values for BMI (at ~26) than non-PCOS patients. PCOS patients were not obese (BMI ˂ 30), but rather overweight. As expected, the PCOS patients showed an antral follicle count (AFC) that was significantly higher than that of the non-PCOS women (56.1 ± 4.02 vs. 19.8 ± 1.10, respectively). The serum levels regarding the LH, anti-Müllerian hormone (AMH) and LH:FSH ratio were significantly higher in the PCOS group than in the control group (*p* ˂ 0.05, *p* ˂ 0.001 and *p* ˂ 0.01, respectively).

### 2.2. Altered Expression of Endocrine-Linked Genes in GCs of Follicles from PCOS Women

**Figure 1:**
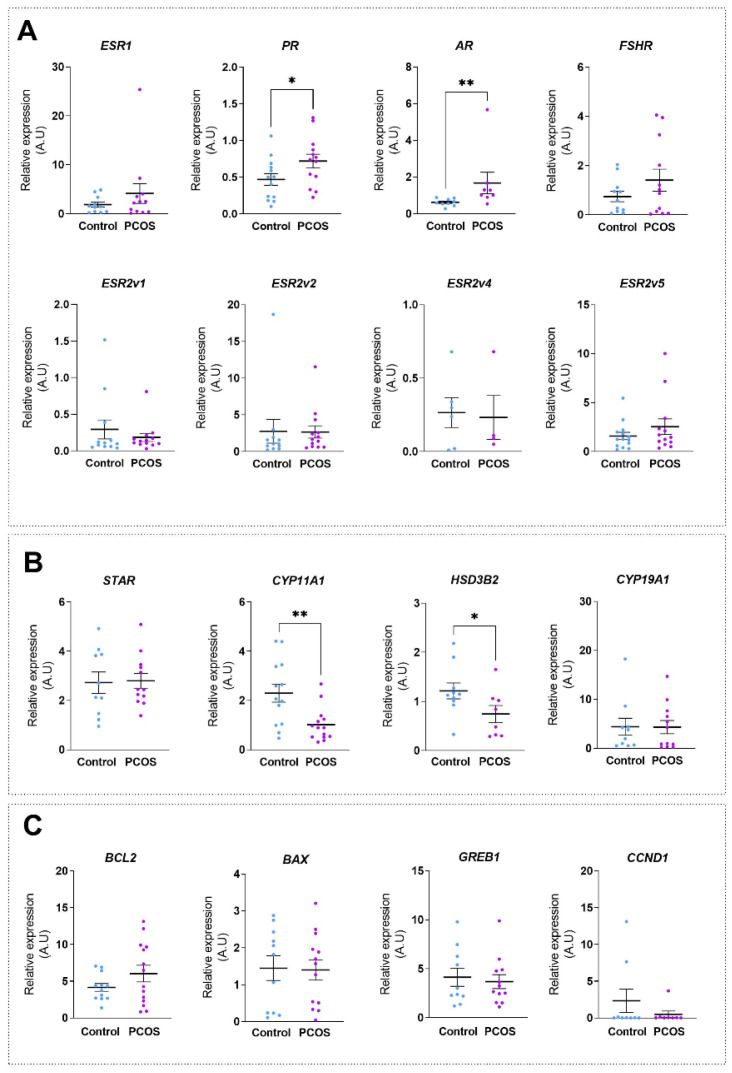
Altered expressions of specific genes in GCs of follicles from PCOS women. Analysis of transcriptional change in genes associated with GC functions in GCs of PCOS and control women. Relative levels of (**A**) steroid receptors *ESR1*, *PR*, *AR*, *ESR2v1*, *ESR2v2*, *ESR2v4, ESR2v5* and *FSHR,* (**B**) steroidogenic enzymes *STAR*, *CYP11A1*, *HSD3B2* and *CYP19A1* and (**C**) growth regulators *BCL2*, *BAX*, *GREB1* and *CCND1* mRNAs were determined via RT-qPCR analysis for each group. Transcript levels were normalized to *GAPDH* transcript abundance. Values are represented as means ± SEM (*n* = 6–14), measured in triplicate. Differences between groups were considered significant for *p* ≤ 0.05 * and *p* ≤ 0.01 ** via Mann–Whitney test. A.U., Arbitrary Units.

First, we compared the gene expression profiles of a subset of genes associated with endocrine cell functions (steroid receptors, steroidogenic enzymes and growth regulators) in GCs from PCOS and non-PCOS control women. The relative expression level of 16 selected genes (including eight target genes of E2 [28,33,34,35,36,37]) were analyzed via RT-qPCR. As shown in Figure 1A, the abundance of mRNA in nuclear estrogen receptors (ERs) (*ESR1*, *ESR2* isoforms *ESR2v1*, *ESR2v2*, *ESR2v4* and *ESR2v5*) was not different between PCOS and control groups, whereas that of the progesterone receptor (*PR*) gene was significantly higher by 1.53-fold (*p* = 0.044) in the PCOS group (Figure 1A). In addition, GCs of PCOS women displayed a 2.68-fold increase (*p* = 0.003) in the androgen receptor (*AR*) mRNA levels, which is in accordance with other studies [38,39,40]. No significant difference was observed between the two groups in the gene expression of the FSH receptor (*FSHR*), a critical mediator of follicle growth (Figure 1A). When considering the steroidogenesis actors (Figure 1B), we showed that the steroidogenic acute regulatory protein (*STAR*) mRNA abundance was not significantly different in the two groups, while the mRNA levels of *CYP11A1* (cholesterol chain cleavage enzyme, catalyzing the conversion of cholesterol to P5) and *HSD3B2* (3β-hydroxysteroid dehydrogenase, catalyzing the biosynthesis of P4 from P5) were reduced by 56% (*p* = 0.008) and 38% (*p* = 0.034), respectively, in PCOS GCs as compared to controls. Conversely, we did not detect a significant difference in the expression of the androgen-to-estrogen converting enzyme, *CYP19A1* (aromatase), between the two groups (Figure 1B). Finally, the mRNA abundance of genes encoding either the proapoptotic gene, *BCL-2-associated X* (*BAX*), or the antiapoptotic gene, *B-cell lymphoma 2* (*BCL2*), were unchanged between the control and PCOS groups (Figure 1C). Additionally, we did not observe a difference between the two groups in the expression of genes encoding proliferation-related genes such as *growth regulating estrogen receptor binding 1* (*GREB1*) and *cyclin D1* (*CCND1*) (Figure 1C). Overall, our analyses revealed that in addition to *AR*, the gene expression of *PR*, *CYP11A1* and *HSD3B2* were deregulated in PCOS women.

### 2.3. E2 Lost Its Ability to Regulate Target Gene Expression in GCs from PCOS Follicles

**Figure 2:**
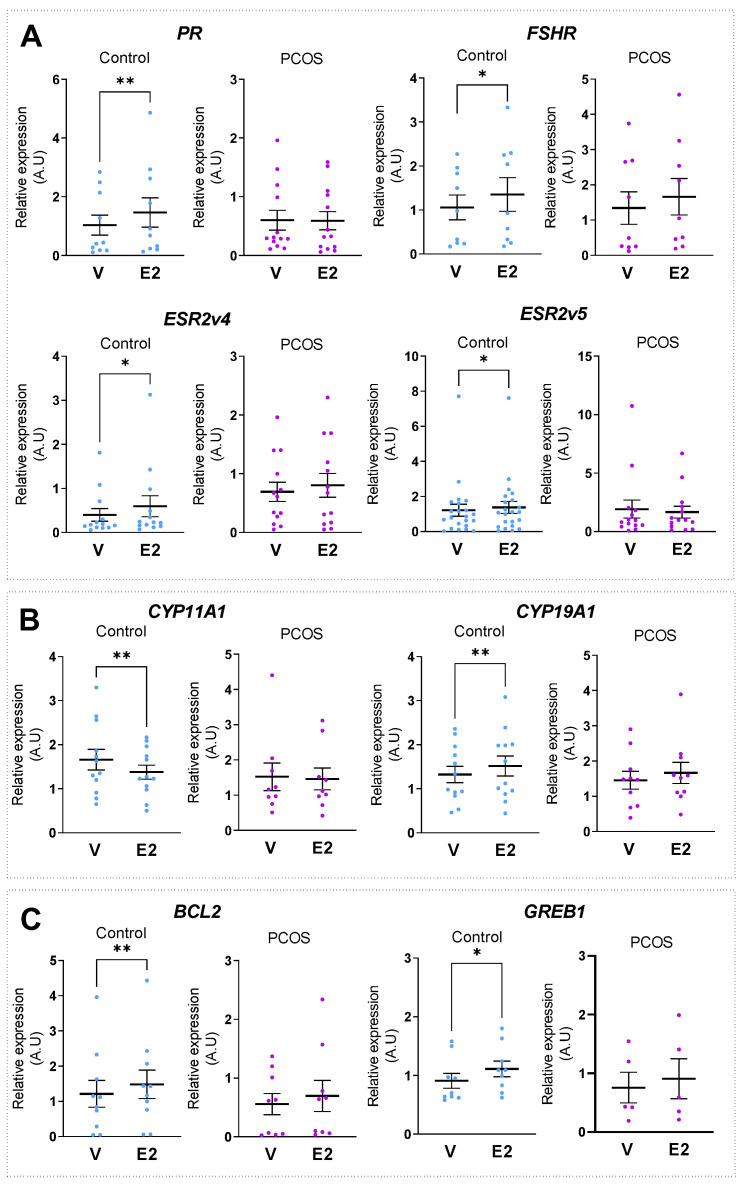
E2 does not regulate E2 target gene expression in GCs from PCOS follicles. Cultured GCs, collected from control and PCOS patients, were treated for 24 h with either vehicle (V) or 10 nM E2. Twenty-four hours later, the relative levels of (**A**) steroid receptors *PR*, *ESR2v4, ESR2v5* and *FSHR* (**B**) steroidogenic enzymes *CYP11A1* and *CYP19A1* and (**C**) cell growth regulators *BCL2* and *GREB1* mRNAs were determined via RT-qPCR analysis for each group. Transcript levels were normalized to *GAPDH* transcript abundance. Values are represented as means ± SEM (*n* = 5–14) from two or three identical wells per patient, measured in triplicate. Differences between treated or untreated groups were considered significant for *p* ≤ 0.05 * and *p* ≤ 0.01 ** by two-sided Wilcoxon signed-rank test. A.U., Arbitrary Units.

Since E2 plays an essential role in follicle growth, as well as in maturation and follicle selection, we compared E2’s ability to regulate the expression of canonical target genes in GCs of control and PCOS women [28,33,34,35,36,37]. For that purpose, GCs were seeded in a steroid-depleted fetal calf serum growing medium for 24 h before a treatment with 10 nM E2 or the vehicle for 24 h. We chose this concentration to reproduce the intrafollicular concentration measured in the FF of the antral follicles [28]. As depicted in Figure 2A–C, a pairwise comparison between the vehicle and E2-treated GCs from control women showed that E2 significantly increased the expressions of gene encoding receptors, such as *PR* (by ~42%; *p* = 0.007), *FSHR* (by ~17%; *p* = 0.014), *ESR2v4* (by ~40%; *p* = 0.010), or *ESR2v5* (by ~14%; *p* = 0.029), when compared to vehicle treatment. We also found that E2 controlled the expression of the steroidogenic enzymes, *CYP11A1* and *CYP19A1* (decrease by ~19% (*p* = 0.005) and increase by ~16% (*p* = 0.007), respectively), as well as those of *BCL2* and *GREB1* (increase by ~26% (*p* = 0.007) and ~24% (*p* = 0.015), respectively) in the control group. By contrast, in PCOS women, the expression of these genes did not change after the E2 treatment when compared to the vehicle condition. Therefore, our data clearly demonstrate that E2 lost the ability to regulate the expression of E2 target genes in PCOS.

**Figure 3:**
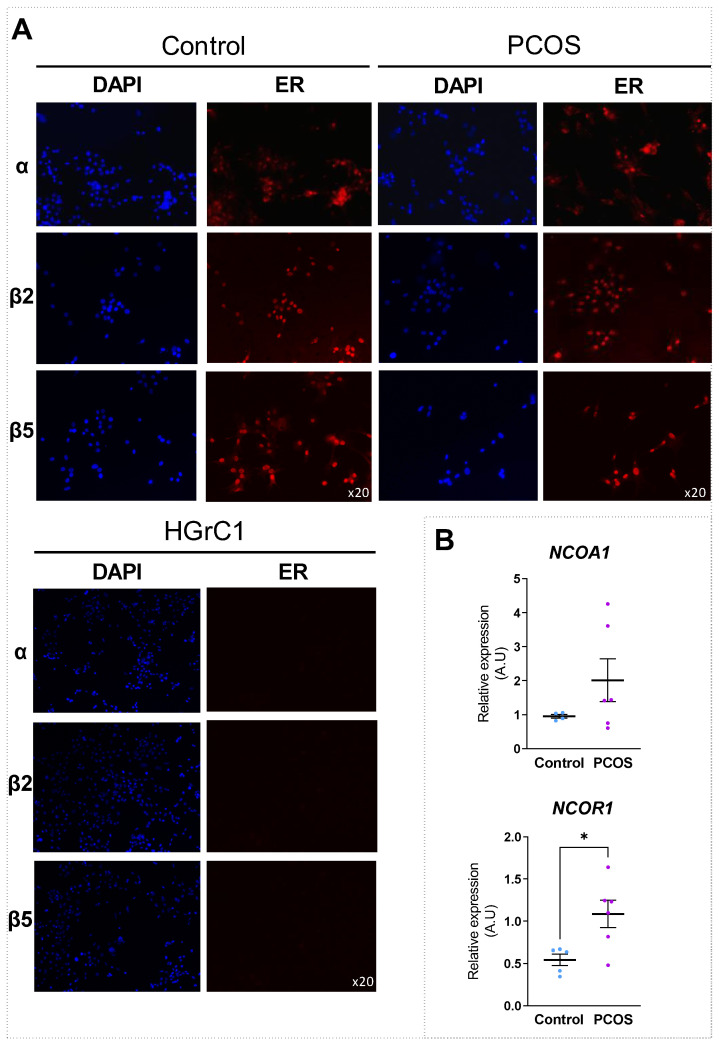
Probable inhibition of ER transactivities in GCs of PCOS. (**A**) GCs of PCOS and control women were fixed and permeabilized, and the localization of ERα, ERβ2 and ERβ5 proteins were monitored via immunofluorescence using anti-ER (α, β2, or β5) antibodies (red). Nuclei were stained with DAPI (blue). Representative images at 20× magnification are presented. The experiment was also performed on HGrC1 cell line as negative control. (**B**) RNA extraction was performed from GCs of PCOS and control women. Relative levels of coregulators, *NCOA1* and *NCOR1* mRNA, were determined via RT-qPCR analysis for each group. Transcript levels were normalized to *GAPDH* transcript abundance. Values are represented as means ± SEM (*n* = 5), measured in triplicate. Differences between groups were considered significant for *p* ≤ 0.05 *, via Mann–Whitney test. A.U., Arbitrary Units.

To understand the molecular basis of E2 inefficiency, we first sought to verify, using immunofluorescence, whether the ERs were properly expressed in the GCs of PCOS. Because of a lack of specific antibodies for ERβ1 and ERβ4, we were able to only follow the protein expression of ERα, ERβ2 and ERβ5 in the GCs of control and PCOS women (Figure 3A). As shown in Figure 3A, ERα, ERβ2 and ERβ5 were all expressed in the nucleus of the GCs of control and PCOS women. The antibody’s specificity was controlled by using the immortalized human granulosa cell line (HGrC1) that does not express ERs [28]. Therefore, the expression of ERs at mRNA levels (Figure 1A) as well as the protein levels for ERα/ERβ2/ERβ5 (Figure 3A) suggests that the absence of E2 signaling in PCOS does not result from an absence of protein expression. Next, to determine whether the estrogen signaling defect in PCOS could originate from a defect in the transcriptional machinery complex, we examined and compared the expression of ER transcriptional co-regulators in GCs of control and PCOS women. Specifically, ER transcriptional activities depend on the presence of coregulators (coactivator or corepressor), which regulate chromatin opening when hormone-bound receptors bind to their target genes [41]. Among them, the best characterized coactivator and corepressor of ERs are NCOA1 (or SRC-1 for steroid receptor coactivator-1) and NCOR1 (nuclear receptor corepressor 1), respectively. We measured their mRNA levels in GCs of control and PCOS women and found a significant up-regulation (by ~99%, *p* = 0.030) of the expression of *NCOR1* in PCOS (Figure 3B), as previously reported [42]. The overexpression of *NCOR1* in PCOS could therefore participate in the inactivation of ER transactivities. We also searched for a role of the NF-κB pathway in E2 inefficiency in PCOS, as previously described in the human ovarian granulosa cell line, KGN [43]. However, the co-treatment of PCOS GCs with an NF-κB inhibitor (BAY11-7082) and E2 did not restore E2 signaling in the GCs of PCOS patients (Supplemental Figure S1).

Taken together, our findings indicate that E2 signaling is impaired in PCOS GCs, possibly by the inhibition of ER transactivities.

### 2.4. Decreased E2 Concentrations in FF of PCOS Follicles Did Not Correlate with Decreased E2 Biosynthesis by GCs

**Figure 4:**
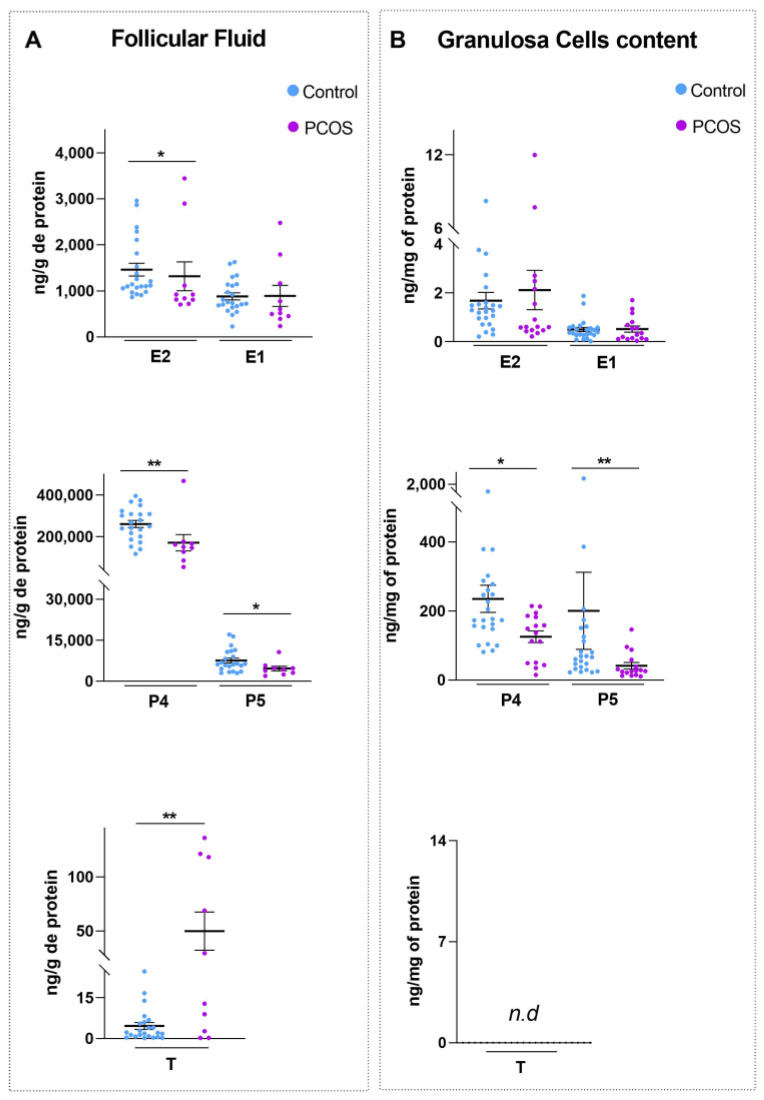
Decreased E2 concentrations in follicular fluid of PCOS follicles do not correlate with decreased E2 biosynthesis. Steroid concentrations of E2, estrone (E1), progesterone (P4), pregnenolone (P5) and testosterone (T) were performed via mass spectrometry coupled with gas chromatography in (**A**) follicular fluid (ng/g of protein) (control, *n* = 23; PCOS, *n* = 10) and (**B**) matched GCs (ng/mg of protein) (control, *n* = 25; PCOS, *n* = 16) of PCOS and control women. Values are represented as means ± SEM. Differences between groups were considered significant for *p* ≤ 0.05 * and *p* ≤ 0.01 ** via Mann–Whitney test. Abbreviations: *n.d*, not detectable.

To study a potential correlation between the concentration of steroids secreted in the FF and that synthetized in the matched GCs, we measured testosterone (T), E2, estrone (E1), progesterone (P4) and pregnenolone (P5) concentrations in these two compartments via mass spectrometry coupled with gas chromatography (GC/MS), which is a highly sensitive and specific method of steroid measurement. As depicted in Figure 4A, the concentration of T in the FF of PCOS was significantly higher (by ~15.5-fold, *p* = 0.008) than that of the control FF, reflecting the local hyperandrogenism of our PCOS cohort. By contrast, E2, P5 and P4 concentrations were significantly lower by ~22.9% (*p* = 0.031), ~38% (*p* = 0.026) and ~29.5% (*p* = 0.012), respectively, in the FF of the PCOS women when compared with that of the control women. Nonetheless, E1 concentration was unchanged between the two groups (Figure 4A). Interestingly, when we compared the concentration of steroids biosynthesized in the GCs of control and PCOS women, we did not observe a decline in E2 biosynthesis in PCOS (Figure 4B), as the FF data might have suggested (Figure 4A). Indeed, as shown in Figure 4B, the measurement of the steroid content in the GCs revealed that E2 and E1 concentrations were not significantly different between the control and PCOS, which is indicative of the normal biosynthesis of both hormones in PCOS. Conversely, both P4 and P5 concentrations were lower in the GCs of PCOS women by ~45.6% (*p* = 0.018) and ~78.6% (*p* = 0.008), respectively, when compared to controls. Finally, T levels were not detectable in the GCs of both groups, possibly because concentrations were below the limit of detection and quantification of GC/MS analysis (≤10 pg).

In conclusion, the measurement of estrogen and progestogen concentration in the FF and GCs of matched follicles highlighted two abnormalities in follicles of PCOS women. Firstly, E2 is similarly biosynthesized in the GCs of control and PCOS women, but its concentration becomes reduced in the FF of PCOS women. Secondly, P5 and P4 concentrations are decreased in the FF of PCOS women, which is probably the result of their reduced biosynthesis in the matched GCs.

## 3. Discussion

**Figure 5:**
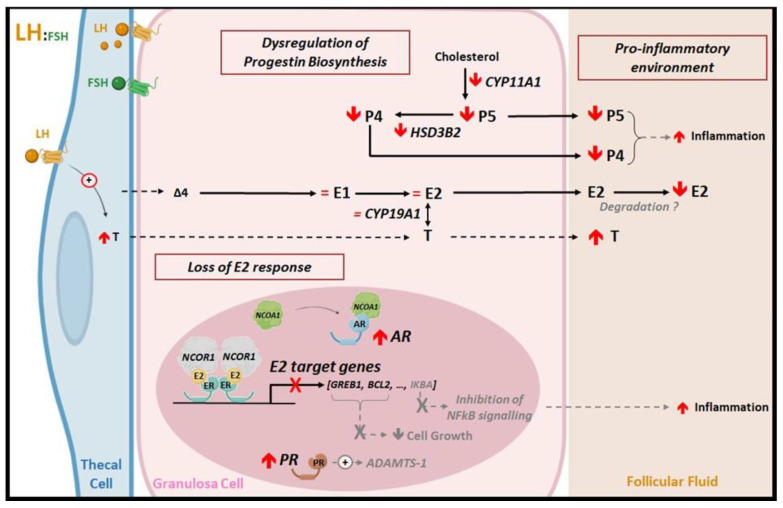
Proposed model of PCOS ovarian context. Our findings demonstrate the deregulation of progestin biosynthesis in the GCs of PCOS that may be responsible for the lower concentrations of pregnenolone (P5) and progesterone (P4) in the follicular fluid (FF). By contrast, the lower level of estradiol (E2) in the FF of PCOS is not associated with a decline in E2 biosynthesis in GCs, but more probably with E2 degradation in the FF. In addition, our study reveals that E2 lost the ability to regulate the expression of E2 target genes in PCOS, such as *GREB1* (growth regulating estrogen receptor binding 1), *BCL2* (B-cell lymphoma-2) or *IKBA* (NF-kappa-B inhibitor alpha). Among the various hypotheses, increased expression of *NCOR1* (nuclear receptor corepressor-1) in PCOS might be implicated in the inhibition of estrogen receptor (ERs) transactivities. In that context, E2 could become unable to control GCs growth and contribute to the inflammatory environment of antral PCOS follicles. ADAMTS-1, a disintegrin-like metalloprotease with thrombospondin type motifs-1; AR, androgen receptor; CYP11A1, cholesterol chain cleavage enzyme; CYP19A1, aromatase; ∆4, delta-4 androstenedione; E1, estrone; ER, estrogen receptor; FSH, follicle-stimulating hormone; HSD3B2, 3β-hydroxysteroid dehydrogenase; LH, luteinizing hormone; NCOA1, or SRC-1 for steroid receptor coactivator-1; NFκB, nuclear factor-kappa B; PR, progesterone receptor; T, testosterone. Black full arrows show new deregulations demonstrated in the study along with known processes; black dashed arrows and grey writing show proposal hypothesis.

In the present study, we provide new insights into the pathogenesis of PCOS by comparing for the first time the concentration of several steroids between the FF and GCs of matched follicles. Indeed, by using a very sensitive, specific and reliable technique (GC/MS), we confirm the lower P4, P5 and E2 concentrations in PCOS FF and demonstrate that only P4 and P5 biosynthesis were altered in GCs of PCOS women, contrary to E2 biosynthesis that was normal in these cells. In fact, when we compared the mRNA abundance of steroidogenic enzymes in GCs of PCOS and control women, we did not observe modifications in the mRNA abundance of *CYP19A1* in PCOS GCs, as reported by others [44], which is in line with the unchanged intracellular E2 content between the control and PCOS groups. Since E2 biosynthesis function is normal in the GCs of PCOS women, our results suggest that the lower concentration of E2 in PCOS FF would probably result from a significant degradation (Figure 5). Although requiring further investigations, this hypothesis is strengthened by the decline in the concentration of E2-binding and E2-protecting proteins such as sex-hormone-binding globulin (SHBG), albumin and heparan sulfate proteoglycan 2/perlecan (another proposed estrogen-binding protein in the FF [45]) in PCOS FF [42,46,47]. Additionally, several studies have shown that FF contains extracellular vesicles, exosomes, that may store proteins and mRNA [16,48]. A proteomic analysis of exosomes was recently performed in human FF from control and PCOS follicles, and a significant difference in protein and mRNA levels was reported [49,50] that may participate in the occurrence of PCOS. Further exhaustive characterization and comprehension of FF exosomes may help to identify proteins that are responsible for E2 metabolization (sulfation or glucuronidation) and/or E2 degradation; we proposed that they play a role in the E2 level decline in PCOS FF.

By contrast, our analyses revealed a decrease in the expression of genes involved in progesterone synthesis (*CYP11A1* and *HSD3B2*) in the GCs of PCOS women. Although we cannot assume that changes in gene expression are associated with equivalent changes in protein expression and function, this result may explain the observed alteration of P5 and P4 biosynthesis in PCOS.

Therefore, our findings demonstrate that the deregulations of steroidogenic activity in the GCs of PCOS women only alter progesterone biosynthesis. As a result, the lower P4 concentration in the PCOS FF may influence the inflammatory environment of the ovaries, since P4 is a pro-inflammatory inhibitor [51] (Figure 5).

Our studies also extend the previous gene profiling analyses of GCs of PCOS [52,53,54]. Indeed, besides an expected up-regulation of the expression of *AR* [38,39,40], our study highlighted a higher mRNA expression of *PR* in the GCs of PCOS women. An increased expression of PR might be the result of the early expression of LH receptors in the GCs of PCOS women [4] along with the higher level of LH secretion [55,56,57]. PR overexpression in PCOS might contribute to the increased levels of ADAMTS-1 (a disintegrin-like metalloprotease with thrombospondin type motifs-1) described in PCOS, and it may participate in the PCOS etiopathology [58,59]. Interestingly, PR overexpression has also been reported in PCOS endometrium [60,61], suggesting the existence of comparable regulations between the ovary and endometrium in PCOS. Furthermore, we did not find a significant modification in mRNA levels of genes encoding FSHR or ERs in PCOS GCs, contrary to other studies [26]. This discordance may reflect differences in PCOS women’s phenotypes and the various IVF stimulation protocols between studies. Indeed, contrary to the majority of IVF protocols, all participants of our study received a GnRH antagonist protocol with recombinant FSH only (no human menopausal gonadotropin) that resulted in comparable levels of serum E2 before hCG induction. Besides, PCOS women were slightly overweight, adding another paradigm that may influence follicles’ characteristics (e.g., increased inflammation environment) and explain the difference between studies. Certain limitations of the present study must be recognized, such as the small number of subjects in the PCOS group when compared to control women, which can be attributed to the rarer nature of the studied PCOS phenotype (D). In addition, the generalizability of the results to all women with PCOS may be limited by the lack of inclusion of women with the more common hyperandrogenic phenotype, as well as the specific body weight nature (overweight but bot obese) of PCOS women examined in this study.

The treatment of GCs with E2 enabled us to further characterize PCOS abnormalities by demonstrating defects in E2 signaling within the GCs of PCOS women. Indeed, we showed that genes encoding PR, FSHR, ERβ2, ERβ5, CYP19A1, BCL2 and GREB1 were up-regulated after the E2 treatment in control GCs, which is in line with effects already described in other species [28,33,34,35,36]. Remarkably, we demonstrated that the E2 treatment did not regulate the expression of *FSHR*, *BCL2* or other target genes in PCOS GCs, like in the control group. As a result, E2 could become unable to control GCs growth in PCOS, since it is inefficient in inducing the expression of the pro-survival *BCL2* gene, as well as the proliferation-inducing gene, *GREB1*. One could assume that E2 inefficiency would also contribute to the inflammatory environment of antral PCOS follicles, as E2 may no longer be able to inhibit TNFα-induced inflammation through ERβ [62] (Figure 5).

This loss of ER transactivity might be caused by several factors. Indeed, a transcriptome analysis of ovaries from PCOS and control women showed that heat shock proteins (HSP90/HSP70) were expressed at a reduced level in PCOS [63]. Heat shock proteins bind to ERs [64] and are necessary to maintain the appropriate conformation required for the hormone-binding activity of the receptor [65]. Therefore, a decline in these chaperones may decrease the amount of ERs that is able to promote their transcriptional activities. Similarly, the lack of ERs signaling in PCOS might result from the increased expression of the ERs corepressor, NCOR1, which blocks ER transcriptional activities. The excess of *NCOR1*, that we and others [42] measured in the GCs of PCOS women, could strongly inhibit ERs transactivation. Additionally, overexpression of *AR* in the GCs of PCOS follicles may have a direct effect on ERs signaling, as reported in a study on breast tumor development showing that the overexpression of ligand-activated AR reduced ERα transactivity by sequestering coactivators like SRC-3/AIB1 [66]. One might speculate then, that higher levels of AR in PCOS could favor NCOA1 sequestration by AR, thereby preventing its interaction with ERs. The potential roles of NCOR1 and AR in the inactivation of E2 signaling in PCOS need to be further evaluated.

In conclusion, our study contributes to the growing body of knowledge on the complex molecular defects in PCOS (Figure 5). Contrary to the current dogma on the alteration of aromatase activity in PCOS follicles, our findings show that aromatase expression and E2 biosynthesis are unaltered in PCOS GCs and rather suggest an increased E2 degradation in matched FF. Notably, we provide a novel hallmark for PCOS by revealing the impairment of E2 signaling in GCs that may contribute to the pro-inflammatory microenvironment of their FF and the deficiency in the emergence of dominant follicles. Further investigations are required to assess the extent of E2-induced gene inefficiency by determining whether the E2 unresponsiveness observed in PCOS GCs could be observed in other E2 target tissues such as endometrium, breast or brain structures (e.g., hypothalamus or pituitary).

## 4. Materials and Methods

### 4.1. Study Participants

Eighty-one women undergoing IVF treatment for infertility at Antoine Béclère Hospital (Clamart, France) were included in this study. Fifty-four non-PCOS women were included as control cohort following the inclusion criteria of infertility due to tubal or male factors. Women with PCOS (*n* = 27) met the following criteria: (1) polycystic ovarian morphology with ovaries containing more than 20 follicles of 2–9 mm in diameter in each ovary, visualized in transvaginal ultrasound scans, and (2) oligomenorrhea (cycle length >35 days) or amenorrhea (cycle >3 months). All patients were aged ≤42 years and had a BMI ˂30 kg/m^2^. Exclusion criteria were also applied to select both PCOS and control patients, i.e., endometriosis, gynecological tumors or presence of premature ovarian failure.

All patients followed a standard IVF gonadotropin-releasing hormone (GnRH) antagonist stimulation protocol. Patients received pre-treatment with oral contraceptive pill or estradiol valerate (2 × 2 mg/per day Provames^®^, Merus Labs Luxco II S, Lëtzebuerg, Luxemburg). Then, recombinant FSH (150–300 IU, Gonal-F^®^, Merck-Serono, Calais, France) was administered from day 3 for at least 5 days at initial dosage, and 0.25 mg GnRH antagonist (Ganirelix, Orgalutran^®^, MSD, Puteaux, France) or Cetrorelix (Cetrotide^®^, Merck-Serono, Calais, France) was systematically started on day 6. From the sixth day of recombinant FSH therapy onwards, daily FSH doses were adjusted according to E2 levels, the number of growing follicles, or both. Due to risks of ovarian hyperstimulation in PCOS, gonadotropin doses could be slightly lower in PCOS than in control group. Nevertheless, we verified that there was no incidence of gonadotropin doses on either serum E2 concentration or oocyte maturation [67]. During the last days of ovarian stimulation, patients had daily visits for ultrasonographic and hormonal examinations to define the appropriate timing for recombinant human chorionic gonadotropin (hCG) administration. Hormonal measurements were performed using commercially available chemo-luminescence immunoassays with an automated Elecsys immunoanalyzer (ECLIA, Roche Diagnostics, Meylan, France) (Table 1). Administration of recombinant hCG (6500 IU Ovitrelle^®^, Merck-Serono, Calais, France) to trigger ovulation was carried out as soon as four or more preovulatory follicles, measuring 16–22 mm in diameter, were observed and E2 levels per preovulatory follicle were greater than 200 pg/mL. This diameter is associated with the presence of a mature oocyte. Indeed, oocyte maturity was assessed under a microscope by visualizing the polar globule within the perivitelline space, which indicates the resumption of meiosis from prophase I to metaphase II. During our study, the oocyte maturity rate was scored at 80% and was not influenced by the size of the punctured follicle. The post-puncture oocyte maturity rate was constantly assessed at the Antoine Béclère IVF center to ensure that the oocyte collection method remained consistent. Follicles were retrieved 36 h after recombinant hCG administration via transvaginal ultrasound-guided aspiration, and purified GCs were cultured for subsequent analyses, as described below.

### 4.2. Ethical Approval

All women were fully counseled. Informed written consent was obtained from all participants. The study was performed in accordance with the Declaration of Helsinki for Medical Research involving Human Subjects (2013 revision). The investigation received the approval of our internal institutional review board, IRB Blefco-IORG0010582, and is registered under number “2021-1”. Date of the first subject enrollment is January 2021.

### 4.3. Human Granulosa Cell Culture and Treatments

After oocyte isolation, FF and human GCs were collected from pooled antral follicles of each patient. Granulosa lutein cells were purified as previously described [67]. Briefly, FF was centrifuged through a one-step density Percoll gradient (*v*/*v*, Dulbecco’s phosphate-buffered saline (DPBS)/Percoll (Gibco, Thermo Fisher Scientific, Les Ulis, France)) at 4000× *g* for 15 min to remove red blood cells. GCs were collected at the interface, washed with DPBS, resuspended with Dulbecco’s modified Eagle medium (DMEM)/nutrient mixture F-12 Ham (DMEM/F12) (1:1) (Gibco, Thermo Fisher Scientific) supplemented with 10% fetal calf serum (Gibco, Thermo Fisher Scientific) and antibiotics (Penicillin-Streptomycin (5000 U/mL) (Gibco, Thermo Fisher Scientific)). GCs were either frozen at −20 °C until assays (reverse transcription quantitative PCR (qPCR) and mass spectrometry coupled with gas chromatography) and/or seeded (after cell counting and viability testing (~80%) via Trypan blue exclusion) at 1.5 × 10^5^ cells per well in 12-well plates (for mRNA expression analysis) in growing media at 37 °C with 5% CO_2_ to expose to E2 treatment.

Twenty-four hours after seeding in 12-well plates, GCs were cultured in Phenol red-free DMEM/F12 (Gibco, Thermo Fisher Scientific) supplemented with 10% charcoal–dextran-stripped fetal calf serum (Sigma-Aldrich, St. Louis, MO, USA) and antibiotics for 24 h. Cells were then treated with either 10 nM 17β-estradiol (Sigma-Aldrich Inc., St. Louis, MO, USA) (stock solutions at 10 mM in ethanol, diluted in the culture medium to the wanted final concentration) or the same volume of ethanol (solvent vehicle, V) for an additional 24 h.

### 4.4. RNA Extraction and Quantitative Real-Time PCR

Total RNA was extracted using Trizol reagent according to the manufacturer’s protocol, and 1 µg was used for complementary DNA (cDNA) synthesis via reverse transcription SuperScript II reverse transcriptase (Invitrogen, Waltham, MA, USA; Thermo Fisher Scientific) using random primers (Promega, Madison, WI, USA) according to the manufacturer’s instructions. qPCR was performed using a standard SYBR premix Taq kit protocol in 384-well plates (list of primers and sequences in Supplemental Table S1) and the LightCycler 480 Instrument (Roche Diagnostics, Meylan, France), as previously described [28]. Quantification of the amount of target gene mRNAs was calculated relative to that of the *GAPDH* (glyceraldehyde-3-phosphate dehydrogenase), a normalizer gene whose expression was not modulated by estradiol treatment. This quantification was expressed as relative units.

### 4.5. Immunofluorescence

GCs were immunostained with primary antibodies against ERα (abcam32063, 1/200 dilution), ERβ2 (MCA2279GA, BIO-RAD, 1/250 dilution) or ERβ5 (MCA4676GA, BIO-RAD, 1/250 dilution) using a standard protocol as described [28]. Cell nuclei were stained with DAPI, and staining was visualized using a fluorescent microscope (Nikon Eclipse 90i, Tokyo, Japan) equipped with an AxioCam HRc (Zeiss, Marly le Roi, France). Different fields of cells were photographed at ×20 magnification.

### 4.6. Determination of Intracellular and FF Steroid Content

The mass spectrometry coupled with gas chromatography procedure was used to determine E1, E2, T, P4 and P5 concentrations in GCs and FF, as described by Giton et al. [68]. Briefly, GCs were homogenized with a FastPrep-24 (MP Biomedicals, Santa Ana, CA, USA), washed with 1-chlorobutane and resuspended in water containing deuterated internal standard working solution (IS). For FF samples, a volume of 200 µL with 10 µL of IS was directly extracted using 3 mL of 1-chlorobutane. The organic phase was collected on conditioned Hypersep SI 500 mg SPE minicolumn (Thermo Scientific). The column and adsorbed material were then washed with ethyl acetate/hexane (6 mL; 1:9). The second fraction containing the steroids of interest was eluted using ethyl acetate/hexane (4 mL; 1:1), and then evaporated at 60 °C to dryness. E1, E2 and T, were derivatized with pentafluorobenzoyl chloride (Sigma-Aldrich). Final extracts were reconstituted in 20 µL of isooctane, and then transferred into conical vials for injection into the gas chromatography system. P4 and P5 were derivatized with 50 µL of heptafluorobutyric anhydride (Sigma-Aldrich) and anhydrous acetone (1:1) mixture. Final extracts were reconstituted in 20 µL of anhydrous n-hexane, and then transferred into conical vials for injection into the gas chromatography system (GC-2010 Plus, Shimadzu, Kyoto, Japan) using a 50% phenylmethylpolysiloxane VF-17MS capillary column (Agilent Technologies, Santa Clara, CA, USA). A TQ8050 (Shimadzu) triple quadrupole mass spectrometer equipped with a chemical ionization source (NCI) and operating in Q3 single-ion monitoring (SIM) mode was used for E1, E2 and T detection, and with an electron impact source (EI) for operating in multiple-reaction monitoring mode for P4 and P5 detection. The linearity of steroid measurement was confirmed by plotting the ratio of the steroid peak response/IS peak response to the concentration of steroid for each calibration standard. Accuracy, target ions, corresponding deuterated internal control, range of detection, low limit of quantification and intra and inter assay coefficient of variation (CV) of the quality control are shown in Supplemental Table S2.

### 4.7. Statistics Analysis

Data are presented as mean ± SEM (*n* is the number of patients, as indicated in Figure legends). Statistical analyses were performed via Mann–Whitney U test for control and PCOS women comparison, or using Wilcoxon test for vehicle and E2 treatment conditions. *p*-value < 0.05 was considered significant. Data and plots were performed with GraphPad-Prism 8.0.2.

## Figures and Tables

**Table 1:** Comparison of age and serum hormone concentrations of PCOS and non-PCOS patients.

		PCOS (*n* = 27)	Non-PCOS (*n* = 54)	*p*-Value
Basal	Age (y)	32.32 ± 0.60	34.4 ± 0.56	0.009 **
	BMI (kg/m^2^)	26.1 ± 0.94	23.7 ± 0.54	0.0273 *
	Serum LH (UI/L)	8.2 ± 1.06	5.4 ± 0.55	0.0175 *
	Serum FSH (UI/L)	6.3 ± 0.29	7.2 ± 0.34	0.0562
	LH:FSH	1.3 ± 0.20	0.8 ± 0.07	0.0028 **
	AMH (ng/mL)	8.5 ± 0.84	2.1 ± 0.15	<0.001 ***
	AFC	56.1 ± 4.02	19.8 ± 1.10	<0.001 ***
	E2 (pg/mL)	44.8 ± 4.97	90.3 ± 20.87	0.1831
Day before hCG administration	E2 (pg/mL)	2527.6 ± 392.15	2219.0 ± 145.13	0.9603

Abbreviations: BMI, body mass index; LH, luteinized hormone; FSH, follicle-stimulating hormone; AMH, anti-Müllerian hormone; AFC, antral follicle count; E2, estradiol; hCG, human chorionic gonadotropin. Data are expressed as mean ± SEM. Differences between groups were considered statistically significant for *p* ≤ 0.05 *, *p* ≤ 0.01 ** and *p* ≤ 0.001 *** via Mann–Whitney test.

## Data Availability

The data are contained within the article or Supplementary Material.

## Supplementary Materials

The following supporting information can be downloaded at https://www.mdpi.com/article/10.3390/ijms242316689/s1.
