# Supplementary material for: Dysfunction of Human Estrogen Signaling as a Novel Molecular Signature of Polycystic Ovary Syndrome
Source: Int J Mol Sci. 2023 Nov 24;24(23):16689. doi: 10.3390/ijms242316689 (PMC10706349; doi:10.3390/ijms242316689)
Supplement: Supplementary file 1 [file ijms-24-16689-s001.zip › Marie_Figure S1.pdf]

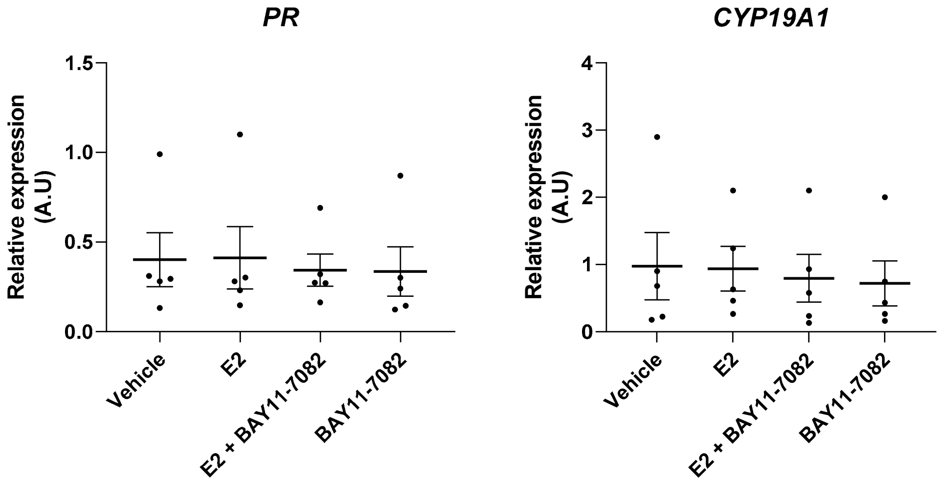

Supplemental Figure S1. NF-κB inhibitor (BAY11-7082) did not restore E2 signaling in GC of PCOS patients. Cultured GC, collected from PCOS patients, were treated for 24 hours with either vehicle, 10 nM E2 with or without 5 μM BAY11-7082 (Calbiochem, La Jolla, CA). Twenty-four hours later, the relative levels of *PR* and *CYP19A1* mRNAs were determined by RT-qPCR analysis. Transcript levels were normalized to GAPDH transcript abundance. Values are represented as means ± SEM (n = 5) from two or three identical wells per patient, measured in triplicate. There was no significant difference between groups of treatment. A.U., Arbitrary Units.
