# Supplementary material for: Dysfunction of Human Estrogen Signaling as a Novel Molecular Signature of Polycystic Ovary Syndrome
Source: Int J Mol Sci. 2023 Nov 24;24(23):16689. doi: 10.3390/ijms242316689 (PMC10706349; doi:10.3390/ijms242316689)
Supplement: Supplementary file 1 [file ijms-24-16689-s001.zip › Marie_Table S1 .pdf]

Table S1. Primers sequences.

**Target Gene (protein)**<br>**Accession no.**	**Forward primer (*Tm*)**<br>**Reverse primer (*Tm*)**	**Product size**
ESR1 (ERα)<br>NM_001122740	5’-CCACCAACCAGTGCACCATT-3’ (59.4°C)<br>5’-GGTCTTTTCGTATCCCACCTTTC-3’ (60.6°C)	116 bp
ESR2 v1 (ERβ1)<br>NM_001437	5’-GTCAGGCATGCGAGTAACAA-3’ (57.3°C)<br>5’-GGGAGCCCTCTTTGCTTTTA-3’ (57.3°C)	192 bp
ESR2 v2 (ERβ2)<br>NM_001040275	5’-TCTCCTCCCAGCAGCAATCC-3’ (61.4°C)<br>5’-GGTCACTGCTCCATCGTTGC-3’ (61.4°C)	162 bp
ESR2 v4 (ERβ4)<br>NM_001214902	5’- GTGACCGATGCTTTGGTTTG-3’ (57.3°C)<br>5’-ATCTTTCATTGCCCACATGC-3’ (55.3°C)	210 bp
ESR2 v5 (ERβ5)<br>DQ838583.1	5’-GATGCTTTGGTTTGGGTGAT-3’ (55.3°C)<br>5’-CCTCCGTGGAGCACATAATC-3’ (59.4°C)	177 bp
GAPDH (GAPDH)<br>NM_002046.7	5’-TCCCTGAGCTGAACG GGA AG-3’ (61.4°C)<br>5’-GGA GGA GTG GGT GTC GCT GT-3’ (63.5°C)	227 bp
GREB1 (GREB1)<br>NM_014668.4	5’-GTGGTAGCCGAGTGGACAAT-3’ (59.4°C)<br>5’-AAACCCGTCTGTGGTACAGC-3’ (59.4°C)	125 bp
CYP19A1 (CYP19)<br>NM_000103.4	5’-CTTTGCCACTGAGTTGATTTTAGC-3’ (59.3°C)<br>5’-ATTAGGGTGCTTTGCAATGAGAAA-3’ (57.6°C)	151 bp
PGR (PR)<br>NM_000926.4	5′-GAAGGGCTACGAAGTCAAA-3′ (54.5°C)<br>5′-GCAGCAATAACTTCAGACATC-3′ (55.9°C)	248 bp

Table S1. Primers sequences.

CCND1 (CyclinD1)<br>NM_053056.3	5′-CCGTCCATGCGGAAGATC-3′ (58.2°C)<br>5′-ATGGCCAGCGGGAAGAC -3′ (57.6°C)	87 bp
BCL2 (Bcl2)<br>NM_000633.3	5′-TCGCCCTGTGGATGACTGA -3′ (58.8°C)<br>5′-CAGAGACAGCCAGGAGAAATCA -3′ (60.3°C)	133 bp
BAX (Bax)<br>NM_001291429.2	5′-TGGCAGCTGACATGTTTTCTGAC -3′ (60.6°C)<br>5′-TCACCCAACCACCCTGGTCTT -3′ (61.8°C)	195 bp
AR(AR)<br>NM_000044.6	5′-CCTGGCTTCCGCAACTTACAC -3′ (61.8°C)<br>5′-GGACTTGTGCATGCGGTACTCA -3′ (62.1°C)	168 bp
CYP11A1 (Cytochrome P450SSC)<br>NM_000781.3	5′-AGCATCAAGGAGACACTAAG -3′ (55.3°C)<br>5′-GCAGGAATCATGTAATCTCG -3′ (55.3°C)	95 bp
FSHR (FSHR)<br>NM_000145.4	5′-GCGGAACCCCAACATCGTGTC -3′ (63.7°C)<br>5′-TGAAGAAATCTCTGCGAAAGT -3′ (54.0°C)	248 bp
STAR (STAR)<br>NM_000349.3	5′-GACAAATGTATGAGTAAAGTGG -3′ (51.1°C)<br>5′-CAGGCTGGTGAGTAATGAATG-3′ (49.7°C)	195 bp
NCOA1 (NCOA)<br>NM_003743.5	5′-ACTTCCAACTCTGCCTCTGC-3′ (59.4°C)<br>5′-TCTTACAGGAGGGTAGCCCC-3′ (61.4°C)	111 bp
NCOR1 (NCOR1)<br>NM_006311.4	5′-TGGACTGACCATGAAAAGGAGA-3′ (58.4°C)<br>5′-GGCAGAAACCAGCAAATTGC-3′ (57.3°C)	197 bp
HSD3 B2<br>*NM_001166120.1*	*Hs01080264_g1*<br>*(Taqman expression probe)*	77 bp
