# Supplementary material for: Dysfunction of Human Estrogen Signaling as a Novel Molecular Signature of Polycystic Ovary Syndrome
Source: Int J Mol Sci. 2023 Nov 24;24(23):16689. doi: 10.3390/ijms242316689 (PMC10706349; doi:10.3390/ijms242316689)
Supplement: Supplementary file 1 [file ijms-24-16689-s001.zip › Marie_Table S2.pdf]

**Table S2. Mass spectrometry coupled with gas chromatography (GC/MS) analytical control validation.**

| **Accuracy** (%) | **Analytes** | **Target (GC-MS) or Precursor ion analyte (GC-MS/MS) / IS*** (m/z) | **Range** (pg) | **Mean** (pg/mL) *Intra- & Inter-assay CVs (%)* | | | |
|---|---|---|---|---|---|---|---|
| | | | | **LLOQ** Mean Intra- & Inter assay CVs | **Low QC** Mean Intra- & Inter assay CVs | **Middle QC** Mean Intra- & Inter assay CVs | **High QC** Mean Intra- & Inter assay CVs |
| 95 - 109 | T / T-d3 | 482 / 485* | 10 - 2 430 | 9.6 | 153.7 | 310.4 | 621.5 |
| | | | | *15.1 - 18.8* | *3.7 - 6.6* | *3.6 - 6.9* | *3.8 - 6.4* |
| 94 - 107 | E2 / E2-d4 | 660 / 664* | 0.2 - 56 | 0.22 | 2.97 | 6.11 | 12.08 |
| | | | | *17.3 - 19.8* | *4.3 - 6.7* | *4.6 - 5.2* | *3.5 - 6.3* |
| 95 - 110 | E1 / E1-d4 | 464 / 466* | 1 - 243 | 1.2 | 25.2 | 48.9 | 100.9 |
| | | | | *18.1 - 19.4* | *6.4 - 8.7* | *5.3 - 7.9* | *4.8 - 6.8* |
| 93 - 110 | P4 / P4-d9 | 510.25>510.20<br>510.25>147.20<br>510.25>495.20<br>519.25>519.20*<br>519.25>147.20* | 20 - 4 850 | 19.6 | 248.8 | 499.1 | 998.3 |
| | | | | *17.4 - 21.3* | *5.5 - 7.4* | *3.3 - 7.8* | *3.5 - 6.8* |
| 93 - 110 | P5 / P5-d4 | 298.30>213.30<br>298.30>145.10<br>298.30>147.20<br>302.30>213.30*<br>302.30>145.10* | 25 - 6 080 | 23.6 | 252.5 | 503.4 | 1007.5 |
| | | | | *19.1 - 23.5* | *6.7 - 8.2* | *5.4 - 7.3* | *4.6 - 7.1* |

*LLOQ: low limit of quantification; QC: quality control*
